# Effects of algal food quality on sexual reproduction of *Daphnia magna*

**DOI:** 10.1002/ece3.2058

**Published:** 2016-03-21

**Authors:** Jong‐Yun Choi, Seong‐Ki Kim, Geung‐Hwan La, Kwang‐Hyeon Chang, Dong‐Kyun Kim, Keon‐Young Jeong, Min S. Park, Gea‐Jae Joo, Hyun‐Woo Kim, Kwang‐Seuk Jeong

**Affiliations:** ^1^ National Institute of EcologySeo‐Cheon GunChungcheongnam Province325‐813Korea; ^2^ Department of Biological SciencesPusan National UniversityBusan609‐735Korea; ^3^ Department of Environmental EducationSunchon National UniversitySunchon540‐742Korea; ^4^ Department of Environmental EngineeringKyung‐Hee UniversityYongin446‐701Korea; ^5^ Department of Physical & Environmental SciencesUniversity of TorontoTorontoONM1C 1A4Canada; ^6^ Institute of Environmental Science & TechnologyPusan National UniversityBusan609‐735Korea; ^7^ Advanced Biomass R&D CenterKorea Advanced Institute of Science & TechnologyDaejeon305‐701Korea

**Keywords:** Algal food quality, *Chlorella vulgaris*, *Daphnia magna*, sexual reproduction, *Stephanodiscus hantzschii*

## Abstract

The objective of our study was to investigate sexual reproduction of *Daphnia magna* associated with mating behaviors and hatching rates, according to different algal food sources. Since a diatom is known to contain more abundant long‐chain poly unsaturated fatty acids (PUFAs), we hypothesized that the diatom‐consuming *D. magna* would exhibit more successful reproduction rates. Upon the hypothesis, we designed three experiments using two algal species, a green alga (*Chlorella vulgaris*) and a diatom (*Stephanodiscus hantzschii*). From the results, we found that the mating frequency and copulation duration increased in the treatment with *S. hantzschii*, resulting in a significant increase of hatching rates of resting eggs. In the other two repetitive mating strategies (e.g., one female vs. multiple males, and one male vs. multiple females), we found that the hatching rates of resting eggs were greater in the *S. hantzschii* treatment. In addition to the mating strategy, male body size significantly increased in the diatom treatment, hence average diameter of penis was also statistically different among the treatments (greater diameter in the *S. hantzschii* treatment). To examine the effect of algal food quality, we estimated quantity of fatty acids in the two algal species. Our result showed that *S. hantzschii* had a higher proportion of long‐chain PUFAs than *C. vulgaris*. Furthermore, a stable isotope analysis revealed that carbon and nitrogen originated from *S. hantzschii* were more assimilated to *D. magna*. In summary, our study manifested that diatom consumption of *D. magna* leads to more successful sexual reproduction. We then discussed how the diatom consumption of zooplankton influences food web dynamics in a freshwater ecosystem.

## Introduction

One of the most important (naturally inherent) objectives of living organisms is to sustain stable population dynamics against extinction. In this regard, a reproduction is the kernel process that directly determines the size of population (Guisande 1993; Swamy et al. 2008). Generally, there are two distinct reproduction modes, i.e., asexual and sexual reproduction. The asexual reproduction allows species to increase their population rapidly without wasting resources on male offspring (Agrawal 2001), but often the clone individuals suffer from incurrence of adaptability to environmental fluctuations. The sexual reproduction compensates the disadvantage by allowing offspring to retain beneficial genetic recombination (Kodric‐Brown and Brown 1987; Hamilton 2009). Some species that are vulnerable to environmental fluctuation, such as *Daphnia*, have both reproductive modes through cyclic parthenogenesis, which allows adaptive response to environmental selection pressure (Grebelnyi 1996; Eads et al. 2007; Nevalainen et al. 2011). These changes may be adaptive adjustments to optimize fitness under the set of conditions experienced by the species (Gliwicz and Guisande 1992; Boersma 1995, 1997).

Daphniids are typical of cyclic parthenogenetic species exhibiting this alternation of reproduction processes. During growing season, parthenogenetic reproduction takes place, resulting in rapid growth of population, while they start to produce male individuals as they are under some environmental pressures (e.g., temperature changes; Macháček et al. 2013) and obtain chance to give genetic diversity to the next generation through genetic recombination (Grebelnyi 1996). In this regard, higher mating success and increased hatching rates maintained to ensure successful transfer of potentially amended fitness to the next generation.

It is thought that the reproductive success, as net growth of *Daphnia* population, would be strongly related to food availability and quality. Although numerous environmental factors, such as predation (Stibor and Lüning 1994) and water physicochemistry (Korpelainen 1986), involve reproductive success, the most fundamental one is the energy within the daphniids’ body, which is largely determined by food characteristics (Lampert and Sommer 1997; Nandini and Sarma 2000). In this regard, two aspects have been emphasized: food availability (Ebert 1993; Lampert 1993) and food quality (Ahlgren et al. 1990; von Elert 2002; Kainz et al. 2004). Despite evidences from numerous reproduction studies (Stemberger and Gilbert 1985; Lampert and Sommer 1997; Nandini and Sarma 2000; Choi et al. 2014), most studies have focused on parthenogenetic reproduction, whereas sexual mode has not been investigated intensively (but see Fink et al. 2011; Koch et al. 2011). Furthermore, those studies emphasized maternal influence, that is, the more maternal individuals are healthy, the larger opportunity of producing next generation can be assured. In contrast, role of male daphniids has not been scrutinized.

We speculate that males would also be responsible for successful sexual reproduction. Females’ characteristics are responsible for their survival rate and fecundity, and trans‐generational effects of food condition has been reported with respect to maternal individuals of the former generation, and food quality was shown to affect the number and size of offspring, that is, offspring survival (recognized as “maternal effect”; Glazier 1992). On the contrary, male daphniids exhibit active behavior in sexual reproduction process, that is, they swim toward their mates after sexing and sometimes they fight against competitors (other male individuals; La et al. 2014). Unfortunately, the influence of food quality on sexual reproduction with regard to males’ characteristics has not been scrutinized. For this reason, we expect that if males are sufficiently active by consumption of high‐quality food algae, their sexual reproduction process would be more facilitated.

We hypothesized that characteristics of daphniids’ sexual reproduction might respond to different algal food source. Typical algal species in freshwaters where daphniids encounter and consume are green algae or diatoms in edible sizes, thus in this background, we used two phytoplankton species, *Chlorella vulgaris* and *Stephanodiscus hantzschii*, as food, and investigated *Daphnia magna* sexual reproduction characteristics in response to different food algal treatments. In the simple mating experiment, we examined mating frequency and copulation duration. Two repeated mating experiments were then implemented: (1) female‐oriented (one female vs. one to 20 males), and (2) male‐oriented (one male vs. 20 females) in order to investigate hatching rates of resting eggs. Also, we examined fatty acid species and quantity of two different algal foods, and determined contribution of algal food to the grazers by means of stable isotope analysis.

## Plankton Culture Maintenance and Preparation

### Plankton subculture

Plankton culture was based on three species: one cladoceran zooplankton *D. magna*, and two phytoplankton species, a green alga *C. vulgaris* (strain number, UMACC 001) and a diatom species *S. hantzschii* (strain number, CPCC 267). We obtained *D. magna* clones from the National Institute of Environmental Research of Korea. The clones were preserved in 500‐mL transparent glass beakers containing Elendt M4 medium (Elendt 1990), and they were stored in a growth chamber (Eyela FLI‐2000; Eyela, Tokyo, Japan) at 20°C, under a 12L:12D light‐dark cycle, 30 photon flux density (*μ*mol·m^−2^·sec^−1^). We provided only *C. vulgaris* as food for *D. magna* during the subculture maintenance period.

The algal species were used as food sources for *D. magna* during the experimental period. They were stored separately in different growth chambers (Chemtopia SDI‐432C chambers; Chemtopia, Seoul, South Korea). To preclude a different quality of algal food associated with temperature in which each species grows well, we cultured both algal species at their optimal temperature conditions, that is, *C. vulgaris* under 27°C; a 12L:12D cycle; and 70 photon flux in Bold Basal Medium (Nichols 1973). *S. hantzschii* tolerates a wide range of temperatures, but favors relatively low temperatures (Swale 1963; Kim et al. 2008; Jung et al. 2011). Thus, it was preserved in a cold environment (10°C; a 12L:12D cycle; 70 photon flux) in Diatom Medium (Beakes et al. 1988).

### Algal food preparation

First, we defined three algal food treatments, that is, STE indicated the experimental group exposed to only *S. hantzschii* provision, CHL indicated only to *C. vulgaris*, and MIX indicated the mixture of algal food.

We determined algal food quantity according to Strathmann (1967). A carbon content of algae approximately equal to 2.5 mg·C L^−1^ in a given volume of zooplankton medium would be sufficient for zooplankton survival and population growth. We used 500‐mL beakers for the entire experiment and the amount of algal food was adjusted to 2.5 mg·C L^−1^. The required amount of algae to supply 2.5 mg·C L^−1^ can be calculated by the relationship between algal density and carbon content (Strathmann 1967), if algal cell size is available. Thus, we obtained size information for *C. vulgaris* and *S. hantzschii* by measuring their diameter 50 times, and calculated the average (Table S1). Based on the size, we calculated the algal cell density to meet the 2.5 mg·C L^−1^ requirement. Algal density changes continuously during cultures, thus the injection volume was determined daily.

We also established a scheme for providing a mixture of the two algal species, according to Choi et al. (2014). We provided the cladocerans with 1.25 mg·C L^−1^ of each of the two algal species, comprising 2.5 mg·C L^−1^ in total. The density of the two mixed species was daily enumerated. We injected algal food between 3:00 pm and 4:00 pm.

## Experimental Designs

**Figure 1:**
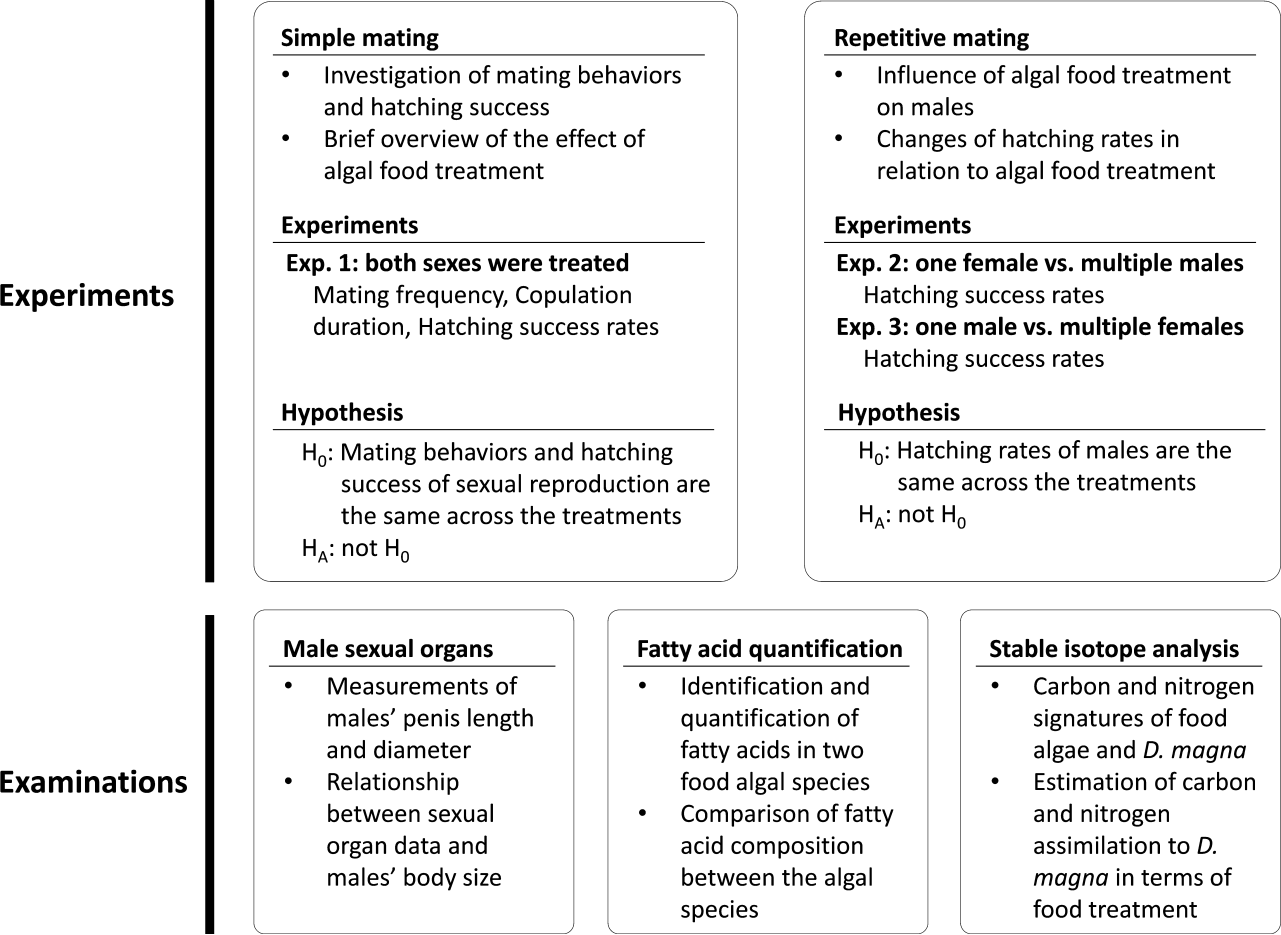
Brief summary of experimental approaches and examinations of the current study.

We utilized males and sexual females in three mating experiments. The preparation methods the sexual individuals are shown in the Data S1. Using the birth‐time synchronized males and sexual females, we implemented the following experiments (see Fig. 1 for summarized information of the current study).

### Experiment 1: sexual reproduction

To investigate the influence of algal food species on mating behavior (mating frequency and copulation duration) and hatching rates of resting eggs, 30 observations (10 observations per each algal food treatment) were conducted during the daytime (10:00 am to 4:00 pm). Three males and three sexual females were allocated in each chamber within each treatment: i.e., a total of 90 males and 90 females (30 males and 30 sexual females per each food treatment) were used.

For the experiment, we used three small acrylic chambers, convenient for two‐dimensional observations (length × height × width = 8.5 cm × 7 cm × 0.5 cm). The observation chambers were placed in three boxes made of black panels without cover and front panel, and a 20‐W fluorescent lamp was placed above each box for illumination. We filled the observation chambers with 30 mL clear M4 medium without a food supply. We placed three digital camcorders (Sanyo Xacti VPC‐SH1, Tokyo, Japan) in front of the chambers (one camcorders per one chamber), and recorded the mating behavior in each chamber at 1280 × 720 resolution (30 frames per second; fps). Simultaneously three observations were made, and a total of 10 observations were implemented per algal food treatment.

We recorded mating activities for approximately 35 min per observation, and the initial 10‐min video clip was discarded to ensure *D. magna* acclimation. The following 15‐min record was used for analysis and the remaining 10 min were not used. Discrimination between mating and simple contact between a male and a female was accomplished in accordance with La et al. (2014), and simple collisions between males or females were excluded. We counted the number of successful mating attempts during 15 min. For every mating event, we recorded the frame number at the initiation and termination points. The total number of frames for each mating was also used to calculate the copulation duration (unit, seconds). We used a video software (GomPlayer, Gretech, South Korea) to observe frame counts. Three experts ran every 15‐min clip to minimize observation errors.

After the mating experiment, we transferred the mated females to six‐well plates, with one individual per well filled with 10 mL clear Elendt M4 medium. The females were incubated at 20°C until they laid resting eggs. Resting eggs were transferred to 12‐well plates (one resting egg per well) filled with clear M4 medium, and they incubated at 15°C. Hatching rates were calculated as follows: number of hatched neonates/number of eggs × 100%. One resting egg sac contains two eggs; therefore, the hatching rates were 0% (no hatches), 50% (one egg hatched), or 100% (both eggs hatched) per replication. If the hatching rates of 10 replicates were not significantly different within a treatment combination, we pooled the number of hatched neonates, and that number was divided by 20 (two eggs per resting egg sac × 10 replications), which was regarded as a pooled hatching rate. The success rates were examined among the three algal food treatments.

### Experiment 2: female‐oriented repeated mating

**Figure 2:**
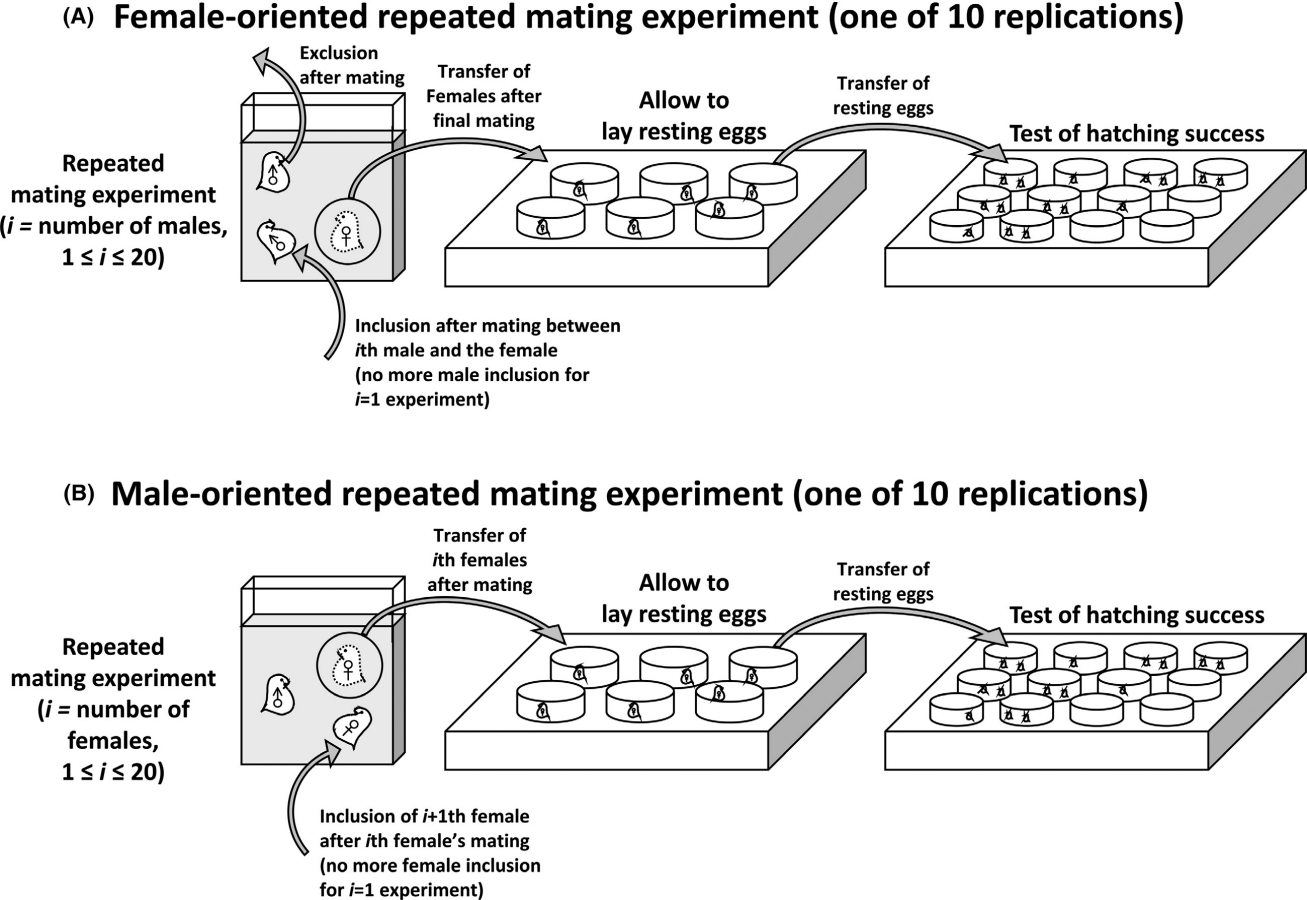
Schematic diagram of the female‐ and male‐oriented repeated mating experiments.

To investigate the influence of mating frequency on hatching success, we implemented two separate experiments: female‐oriented (i.e., one female mated multiple males) and male‐oriented (one male mated multiple sexual females; see Fig. 2A). The concept of the female‐oriented experiment was as the follows: if one sexual female *D. magna* mated with more number of males, then hatching rates would increase relative to mating frequency. In addition, the influence of food treatments was simultaneously observed.

In this experiment, the maximum mating frequency (hereafter, “mating frequency” of the repetitive mating experiments is shown as MF) was set up to 20. For example, once a sexual female at the first trial (i.e., MF1) successfully mated with one male, the corresponding male was immediately removed from the chamber. Then, the female was conveyed to a well‐plate in order to obtain its resting egg. At MF2 trial, a new sexual female was experimented to allow mating with two male individuals in a row. Likewise, the first male was immediately removed after mating, and subsequently a new male was introduced to the female for the second mating. After the final mating event (in the case of MF2, the mating with the second male), we transferred the mated female to a well‐plate. In doing so, all the mating experiments were conducted up to MF20. For all MF1 to 20 experiments, we never re‐used any of male individuals during the experiment, and also we used different sexual females for each of MF experiments. Discrimination between mating and simple contact between a male and a female was accomplished in accordance with La et al. (2014). Every MF experiment had 10 replications.

As summarized, the factors we controlled were (1) three treatments of food algae (i.e., CHL, STE, and MIX) only applied to males, and (2) the number of males (1–20), resulting in 3 × 20 = 60 cases. Every case had 10 replications (=total 600 mating experiments); therefore, we prepared a total of 600 sexual females (three food treatments × 20 mating attempts × 10 replications) and 6300 males (three food treatments × (sum(1:20) males = 210 males) × 10 replications) for the experiments. The chambers used in the simple mating experiment (i.e., the experiment for investigating mating frequency and copulation duration) were used in this experiment.

Consequently, we obtained a total of 600 mated sexual females from the female‐oriented mating experiment, and they were transferred to six‐well plates, one mated female per well, as described above. Every well was filled with 10 mL clear Elendt M4 medium, and the females were incubated at 20°C until they laid resting eggs. The resting eggs were transferred to 12 well plates (one resting egg per well) filled with clear medium and incubated at 15°C for 6–13 days until they hatched. Hatching rates were calculated in the same way to the sexual reproduction experiment. The success rates were examined in comparison of three food treatments and number of male individuals.

### Experiment 3: male‐oriented repeated mating

The male‐oriented hatching success experiment was to examine vigor of males in accordance with different food algal treatments. We designed the experiment as follows: one male individual was repeatedly allowed to mate with 20 sexual females sequentially (one sexual female at one mating attempt; Fig. 2B). The mated sexual female was removed after mating, and then a new sexual female was introduced. A total of 10 replications per food treatment were used.

For this experiment, we prepared a total of 30 male *D. magna* individuals (10 replications × three food treatments) and 600 sexual females (10 replications × 20 mating attempts × three food treatments). Every sexual female mated with male individuals were transferred to six well plates (one female in one well) in order to let them lay their resting eggs. The remaining protocol for hatching success evaluation was identical to the female‐oriented experiment (i.e., Experiment 2) including the experiment chambers.

### Statistical analysis

For the statistical comparison of the experimental groups, we used a one‐way, nested analysis of variance (two‐tailed, *α *= 0.05) for data from sexual reproduction (mating frequency and copulation duration). Even though, we prepared 10 replicates (i.e., beakers) for every experimental group, the pseudo‐replication problem had to be carefully considered (Hurlbert 1984; Choi et al. 2014). Therefore, we regarded the different food treatments as the primary factor, and 10 beakers as nested subgroups for every treatment.

To determine trend of hatching rates after female‐ and male‐oriented repeated mating experiments, we implemented a regression analysis to the hatching rate data. All statistical tests were conducted using the statistical package SPSS Statistics ver. 20 (IBM, New York, USA).

### Male penis characteristics

**Figure 3:**
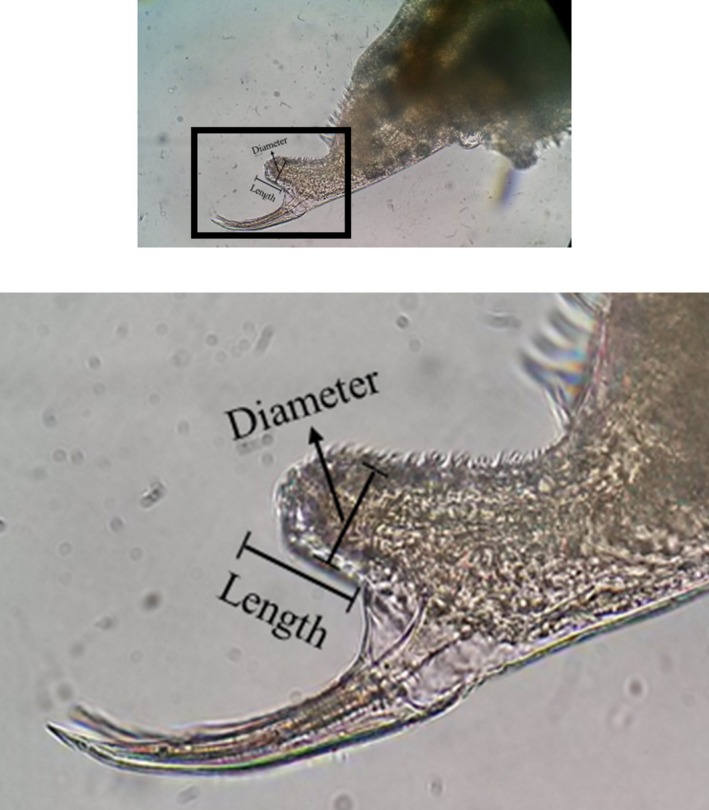
An image showing the length and width of male *Daphnia magna* penis.

We collected the males used in the mating experiment after termination of the experiment, and stored them in ethanol. Using a microscope, we obtained the image of the males in order to measure their body length (×400 magnification, AxioScope 40; Carl Zeiss Microscopy, Germany). Then, we eviscerated penises under the microscope, and took microscopic images of the penis samples as well. Body length, size of penises (width and length) were measured using an image processing program (AxioVision Rel 4.8; Carl Zeiss Microscopy; Fig. 3), and the measurements were compared with respect to food algal treatments.

### Stable isotope analysis

The 30 adult *D. magna* in each experimental group and the two algal subcultures were used for stable isotope analysis. The *D. magna* samples contained phytoplankton in their guts; therefore, we transferred these individuals into fresh Elendt M4 medium for more than 24 h without provision of additional food. This allowed these individuals to eject their gut contents; they were then included in the stable isotope analysis. The 30 *D. magna* individuals were divided into six groups (*n* = 5 per group); three of these groups (*n* = 15 individuals) were used for detection of the carbon signature and three groups were used for detection of the nitrogen signature.

Carbon and nitrogen measurements from *D. magna* were conducted separately. It is necessary to extract tissue lipids for accurate interpretation of trophodynamics using stable isotope data for carbon. The carbon isotope signature depends on protein content in tissue; the presence of lipids can affect the reliability of the isotope analysis. Lipid content varies in accordance with tissue type and is ^13^C‐depleted relative to proteins. Therefore, tissue samples that contain lipids may produce an unstable carbon isotope signature. In contrast, lipid extraction affects *δ* ^15^N. Therefore, we divided the samples into separate groups for carbon‐ and nitrogen‐signature analysis. Lipids were removed only from the carbon‐signature samples. Comparison between the two samples was accomplished by *δ* ^13^C and *δ* ^15^N analyses (Post et al. 2007). The carbon‐signature samples were placed in a solution of methanol‐chloroform‐triple‐distilled water (2:1:0.8 v/v/v) for 24 h.

For stable isotope analysis of food algal subcultures, we prepared 5 mL of algal suspension from each species and analyzed them in triplicates. The algal samples were treated with 1 mol hydrochloric acid (HCl) to remove inorganic carbon. The samples were then rinsed with ultrapure water to remove the HCl.

The prepared samples (two algal species and *D. magna* from three treatments) were freeze‐dried and then ground with a mortar and pestle. The powdered samples were maintained at −70°C until analysis. When all samples were collected, carbon and nitrogen isotope ratios were determined using continuous‐flow isotope mass spectrometry. Dried samples (approximately 0.5 mg of animal samples and 1.0 mg of algae) were combusted in an elemental analyzer (Euro EA 3000, EuroVector, Milano, Italy), and the resultant gases (CO_2_ and N_2_) were introduced into an isotope ratio mass spectrometer (CF‐IRMS, model‐ISOPRIME 100; Micromass Isoprime, GV Instruments Ltd., Manchester, UK) in a continuous flow, using helium as the carrier gas. Data were expressed as the relative per‐mil (%) difference between sample and conventional standards of Pee Dee belemnite carbonate for carbon and atmospheric N_2_ for nitrogen, according to the following equation:

$$\delta \;X\left(‰\right)=\left[\frac{R_{\mathrm{sample}}}{R_{\mathrm{standard}}}-1\right]\times 1000,$$

where *X* is ^13^C or ^15^N, and *R* is the ^13^C:^12^C or ^15^N:^14^N ratio. A secondary standard with a known relationship to the international standard was used as a reference material. The standard deviations of *δ* ^13^C and *δ* ^15^N for 20 replicate analyses of the “Peptone (*δ* ^13^C = −15.8‰ and *δ* ^15^N = 7.0‰, Merck)” standard were ±0.1 and ±0.2 (‰), respectively.

To determine which of the potential food sources in the mixed culture of *Chlorella* and *Stephanodiscus* was predominantly assimilated by *D. magna*, we estimated the proportional contribution of each food source using a mixing model with the SIAR package for R Statistics software ver. 3.0.2 (Parnell et al. 2010; R Core Team 2013). We conducted modeling using default parameters (interactions = 500,000; burnin = 50,000; thinby = 15). Fractionation factors (average ± SD) used for *D. magna* were 0.5 ± 0.2‰ for Δ^13^C and 3.0 ± 0.5‰ for Δ^15^N (Syväranta et al. 2011) because we assumed that the values showed no variation depending on the trophic position (Vanderklift and Ponsard 2003).

### Fatty acid analysis for microalgal species

In order to examine fatty acid composition of the two microalgae, we prepared 12 subcultures as follows: two temperatures (10 and 27°C) × two species (*S. hantzschii* and *C. vulgaris*) × three replicates, under the identical culture conditions for algal growth. The incubation period was 28 days. For the diatom species, 1000 mL Diatom Medium was used per replicate and the same volume of Bold Basal Medium was used for the green algae.

At the 28th incubation day, we centrifuged every culture using centrifugation tubes, at 4000 rpm, at 4°C for 10 min. The supernatant was carefully removed, and the remaining pellet was transferred to a 1.5‐mL sterile tube. Liquid nitrogen was used to flash freeze the samples and they were stored at −80°C until further fatty acid analysis.

A known amount (approximately 10 mg) of dried algal cell biomass was used for lipid extraction using 2 mL of 2:1 chloroform and a methanol mixture in Teflon‐sealed, screw‐capped Pyrex tubes for 10 min followed by vortexing. One milliliter of chloroform containing 0.5 mg of heptadecanoic acid (C17:0) was added to each tube as an internal standard, and 1 mL of methanol and 300 *μ*L of sulfuric acid were added for transesterification reaction under 100°C for 20 min using a WiseTherm HB 96‐D heating block (WiseTherm, Seoul, Korea). After the reaction, the samples were cooled to room temperature, and 1 mL of deionized water was added to wash out residual methanol and sulfuric acid. The samples were then centrifuged for phase separation, and the lower chloroform phase was separated and filtered through a 0.2 *μ*m PVDF syringe filter (Whatman, Sprinfield Mill, UK) prior to gas chromatography (GC) analysis. GC analysis for all samples was carried out using an Agilent 6890 Gas Chromatography (Agilent, Santa Clara, CA) equipped with a flame ionized detector and HP19091N‐213 HP‐INNOWax polyethylene glycol column (Agilent). Each FAME peak was identified and quantified with reference to a 37 component FAME standard mix (Supelco, Bellefonte, PA), and the total lipid amount was calculated by summing up all peaks except for the internal standard and solvent peaks.

## Results

### Mating frequency and hatching rates

**Figure 4:**
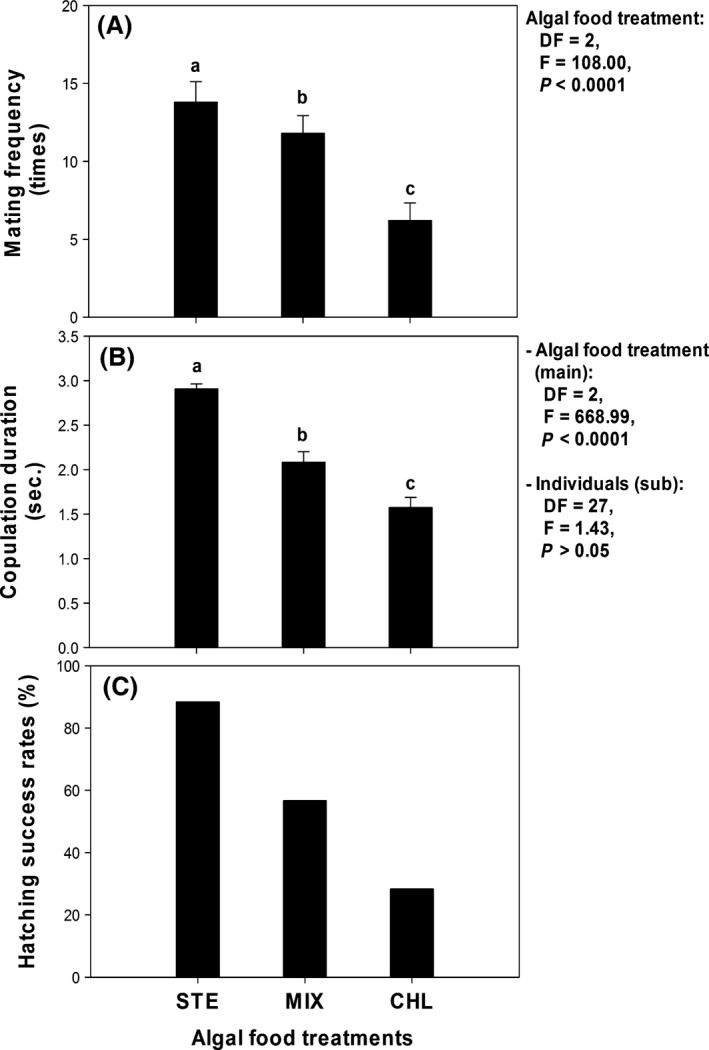
Response of sexual reproduction characteristics to different algal food treatments. A, mating frequency; B, copulation duration; C, hatching rates of resting eggs. Lower case letters indicate statistically different groups determined by post hoc tests.

Mating characteristics were distinguishable with respect to the food treatments (Fig. 4), and diatoms provision significantly increased the mating activity of *D. magna*. Those that consumed *S. hantzschii* attempted mating vigorously during the experiment, and their resting eggs were more viable. The highest mating frequency was observed from the STE group, which was approximately twofold greater than the *D. magna* individuals that consumed only *C. vulgaris* (Fig. 4A). Those in the MIX group attempted mating at an intermediate rate. MF was significantly different among the three groups (one‐way nested ANOVA, Food, *F* = 108.000, df = 2, *P* < 0.001).

Copulation duration was also affected by the food treatments, similar to mating frequency (Fig. 4B). *D. magna* individuals that consumed diatoms exhibited a longer duration of mating than those in the other groups, with the time being twofold greater in comparison with the CHL group. *D. magna* in the MIX group mated for an intermediate length of time. A one‐way, nested ANOVA revealed copulation duration was significantly different among groups (one‐way nested ANOVA, Food, *F* = 668.997, df = 2, *P* < 0.001).

The pooled hatching rates of resting eggs were similarly increased by diatom intake (Fig. 4C). More than 80% of the resting eggs collected from the STE group successfully hatched, but those from the CHL group exhibited a very low hatching rate (approximately 28%). The MIX group exhibited intermediate hatching success, similar to other characteristics.

### Female‐ and male‐oriented hatching success

**Figure 5:**
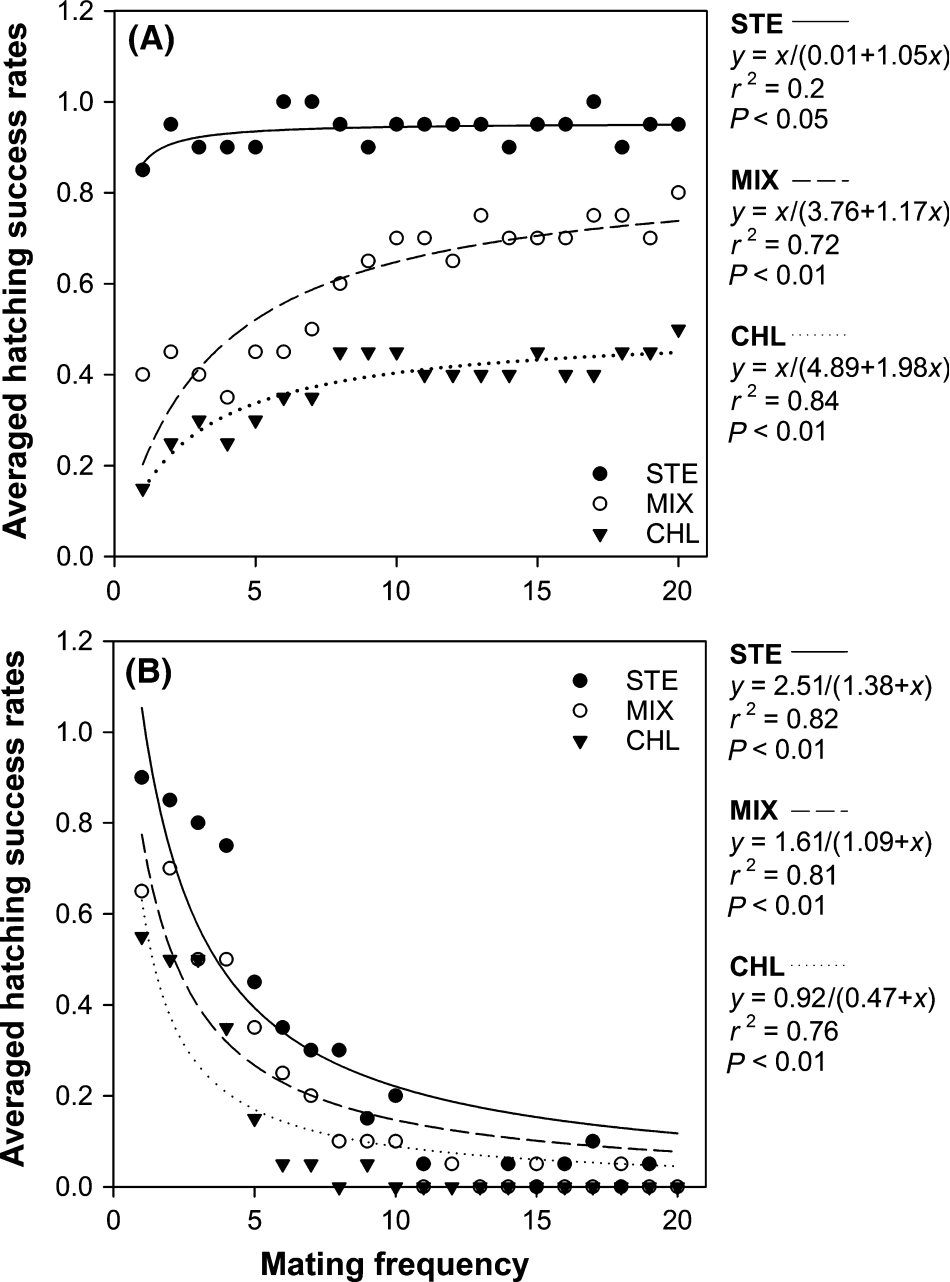
Results of female‐ and male‐oriented repeated mating experiments. A, Female‐oriented hatching rates; B, male‐oriented hatching rates. Solid circles, STE group; Opened circles, MIX group; solid triangle, CHL group. Error bars indicate standard errors (*n *= 10 per each point).

Clear patterns of differences in hatching success were observed from the female‐and male‐oriented experiment (Fig. 5). Hatching rates of sexual females that mated with male *D. magna* from the STE group were steadily maintained at an average of ca 0.9, whereas the hatching rate from CHL group was at an average of ca 0.38 (Fig. 5A). MIX group exhibited an intermediate hatching rate (ca 0.6%). All three groups showed gradual increasing patterns of hatching rate as mating frequency increased. However, the hatching rate of STE group reached its plateau earlier than other groups.

The supply of *S. hantzschii* to male *D. magna* strongly affected longevity of hatching rates, which means that the supply of *S. hantzschii* assured higher hatching success (Fig. 5B). An interesting phenomenon was that the male *D. manga*, which consumed *S. hantzschii* sustained a relatively higher hatching rate than other groups. When we compared the rates at 10th female induction, male individuals in the CHL group could not successfully fertilize resting eggs.

### Male body size according to algal treatments

**Figure 6:**
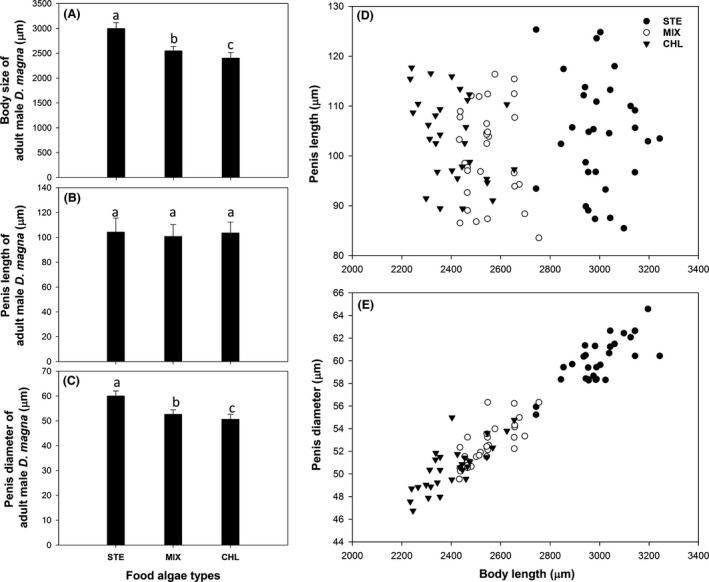
Body and penis morphology of male *Daphnia magna* with respect to food algal treatments. A, body length; B, penis length; C, penis diameter; D, relationship between body length and penis length; E, relationship between body length and penis diameter. Lower characters (a, b, c) in the panels A to C are the statistical subgroups defined by post hoc test.

Male *D. magna* exhibited clear morphological differences in body size and sexual organ except for penis length, along with food algal treatments (Fig. 6). The body size of males was the largest in the STE group, whereas was the smallest in the CHL group (one‐way nested ANOVA, Food, *F* = 248.922, df = 2, *P* < 0.001; Fig. 6A). There was no differences of body length within subgroups (i.e., chambers, *F* = 0.976, df = 27, *P* > 0.05), and the averages of body length among three food algal treatments were different between each other.

Average penis length of male *D. magna* did not show statistical difference between food treatments (one‐way nested ANOVA; Food, *F* = 1.126, df = 2, *P* > 0.05; chamber, *F* = 1.258, df = 27, *P *> 0.05; Fig. 6B), while penis diameter differed along food treatments (one‐way nested ANOVA; Food, *F* = 204.207, df = 2, *P* < 0.001; chamber, *F* = 1.221, df = 27, *P* > 0.05; Fig. 6C). The penis of males was the thickest in STE group, and was the thinnest in CHL group. The average thickness was statistically different along food treatments (post hoc test, *P* < 0.001).

Because all the subgroups (i.e., chambers) were statistically identical in every morphological characteristic, we pooled those data and implemented comparison between body length, penis length, and penis diameter. The body length and penis length did not show a clear relationship (Fig. 6D), while the penis diameter tended to increase as the body length of male *D. magna* became larger (Fig. 6E).

### Stable isotope analysis

**Figure 7:**
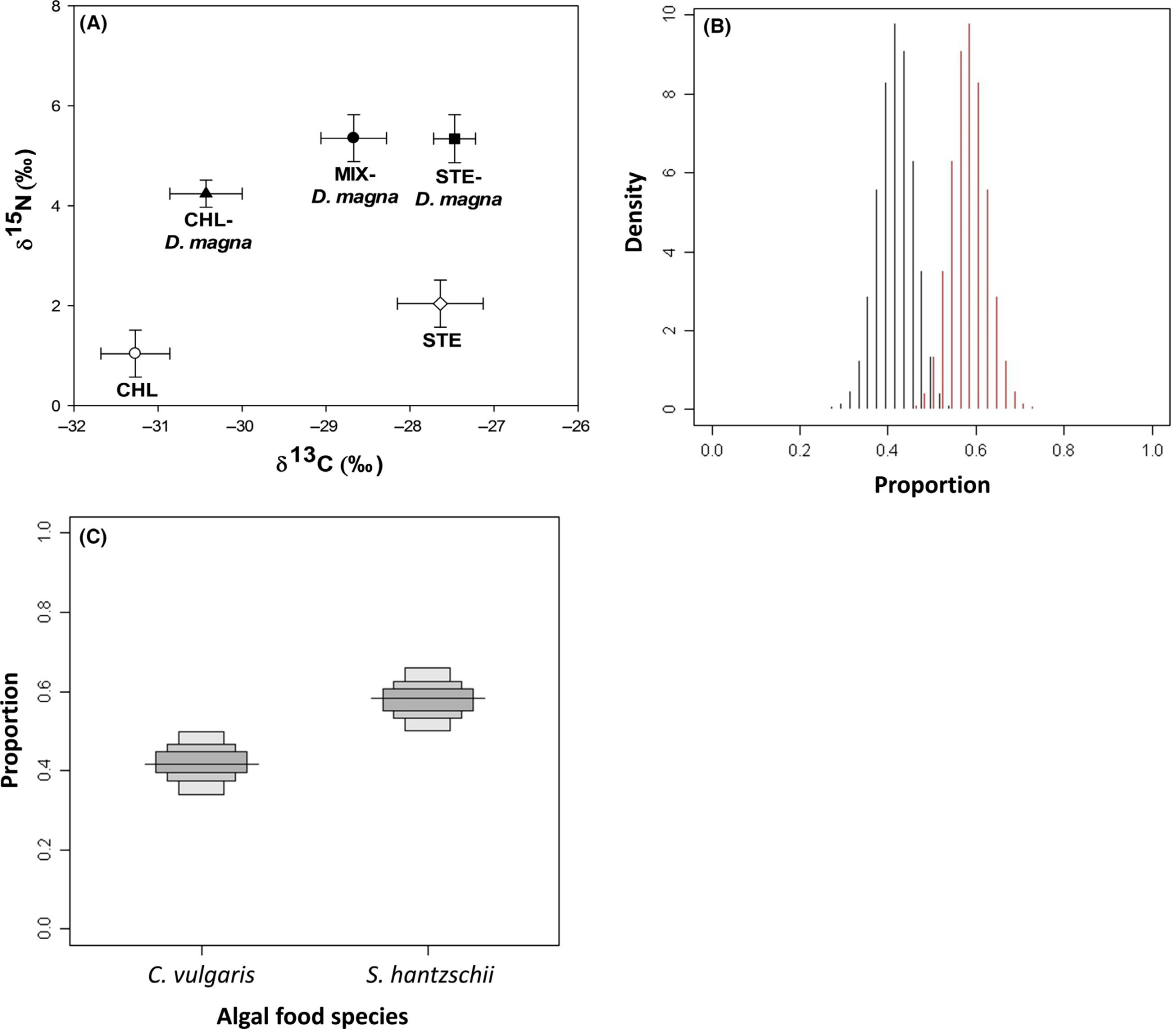
Results of stable isotope analysis. A, *δ* ^13^C and *δ* ^15^N ratio of the algal food sources and *Daphnia magna* in three experimental groups; B, distribution of the solutions for contribution of two resources to the diet of *D. magna* in the SIAR mixing model; C, estimated source proportions (0–1) of *C. vulgaris* and *S. hantzschii* for *D. magna*. Values were calculated using isotopic mass balance (*δ* ^13^C and *δ* ^15^N) for each food source. Lines and boxes indicate averages, and 50%, 75%, and 95% Bayesian credibility intervals for means.

Stable isotope analysis revealed that *D. magna* depended more on *S. hantzschii* than on *C. vulgaris* when they fed on a mixture of these algae (Fig. 7). The *δ* ^13^C and *δ* ^15^N ratio indicated the contribution of phytoplankton eaten by *D. magna*. Some adults fed on only one species (i.e., CHL and STE); either *C. vulgaris* or *S. hantzschii* (Fig. 7A). However, *D. magna* in the MIX group relied more on *S. hantzschii*. Between the two foods, the greater nutritional resources for *D. magna* were in *S. hantzschii* as estimated using SIAR mixing models. Figure 7B shows the model results for the MIX group as a series of stacked histograms that indicated the posterior probability density distributions predicted for the proportions of each food species. The graphic output of the distribution showed separate density distributions for the two food sources with a higher proportion of *S. hantzschii*. In addition, when the contribution rates of the two algal food species in the MIX group were calculated, the *S. hantzschii* contribution rate (ca 60%) was higher than that of *C. vulgaris* (ca 40%) with a narrow, 95%, credibility interval (CI) indicating a very high degree of accuracy (Fig. 7C).

### Fatty acids of food algae

**Figure 8:**
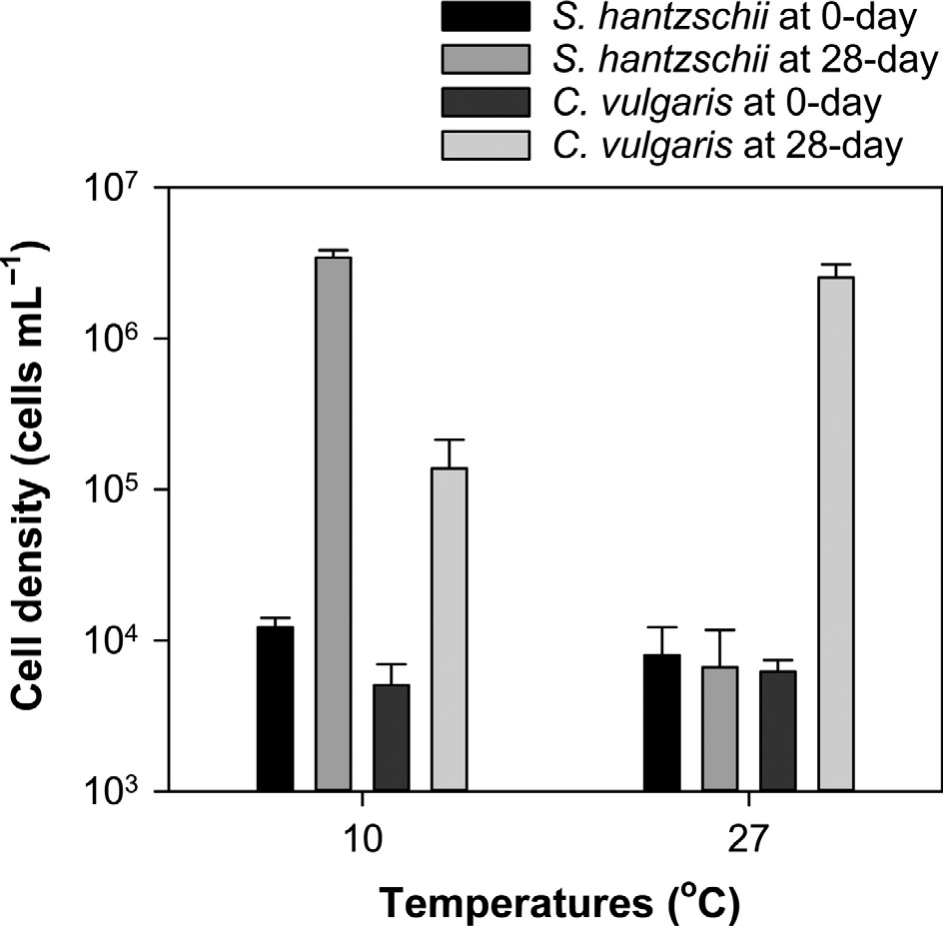
Cell density measurements for *Chlorella vulgaris* and *Stephanodiscus hantzschii* incubated for 28 days at different temperatures.

Among four groups of algal cultures, we failed to harvest two groups by the end of growth experiment. Groups under favorable temperature grew well (i.e., *S. hantzschii* at 10°C and *C. vulgaris* at 27°C), and the other two groups (i.e., *S. hantzschii* under 27°C and *C. vulgaris* under 10°C) did not grow (see Fig. 8). Hence, fatty acid quantification was applied to the harvested groups.

Some quantitative characteristics of biomass and lipid contents in two harvested sample groups are summarized in Table 1. Between the two microalgal species, distinct differences in lipid proportion per unit biomass were observed. Lipids in *S. hantzschii* were approximately 28% per unit biomass, whereas those in *C. vulgaris* were approximately 16%.

**Table 1:** Quantitative characteristics of two different food algal species. Every species consisted of three replicates. Numbers within brackets in Food algal species column indicate standard errors

Characteristics	Algal food species		Statistics		
	*Chlorella vulgaris*	*Stephanodiscus hantzschii*	df	*F*	*P*
Total biomass (mg)	4.61 ± 1.76 (1.02)	2.67 ± 1.47 (0.85)	1	2.14	0.22
Total lipid contents (mg)	0.73 ± 0.29 (0.17)	0.74 ± 0.39 (0.23)	1	0.74	0.97
Total lipid proportion to total biomass (%)	15.79 ± 0.19 (0.11)	28.32 ± 2.27 (1.31)	1	90.66	0.001

A total of 22 fatty acids were identified from the GC application, and the composition of fatty acids in the two algal food species was different (Table 2). *S. hantzschii* contained all 21 fatty acid types, whereas *C. vulgaris* possessed 15 types of fatty acids. The abundant fatty acids were C16:0 and C16:1 for *S. hantzschii*, and C16:0 and C18:1n9t,c for *C. vulgaris*. Compared with *C. vulgaris*,* S. hantzschii* contained more of long‐chain Poly‐Unsaturated Fatty Acids (PUFAs). For example, the average proportion of eicosanoic acid (C20:0) was almost similar, but the proportion of eicosapentaenoic acid (EPA; C20:5) was almost 10‐fold greater in *S. hantzschii* than in *C. vulgaris*. Alpha‐linolenic acid (C18:3n3) and paullinic acid (C20:1) were detected only from *S. hantzschii*. In contrast, gamma‐linolenic acid (C18:3n6) was only found in *C. vulgaris*.

**Table 2:** Average fatty acid composition (%) in total lipid contents in two different algal species grown under respective favorable temperature condition

Fatty acids	*Stephanodiscus hantzschii*			*Chlorella vulgaris*		
	Mean	SD	SE	Mean	SD	SE
C6:0	10.0	7.1	4.1			
C8:0	2.0	1.6	0.9			
C10:0	0.1	0.2	0.1			
C12:0	0.2	0.2	0.1	0.1	0.1	0.0
C13:0	1.2	0.6	0.3	0.5	0.3	0.2
C14:0	7.9	1.4	0.8	0.7	0.1	0.1
C14:1	0.1	0.3	0.1	0.3	0.0	0.0
C15:0	0.6	0.1	0.1	0.1	0.1	0.0
C15:1	0.9	0.8	0.4	0.1	0.1	0.0
C16:0	30.7	4.4	2.6	40.4	5.7	3.3
C16:1	28.2	11.5	6.6	5.4	1.3	0.8
C17:0	1.5	1.3	0.8	2.1	1.3	0.8
C17:1	0.4	0.2	0.1	0.1	0.2	0.1
C18:0	3.7	0.1	0.1	18.5	5.4	3.1
C18:1n9t,c	0.8	0.2	0.1	25.0	5.1	3.0
C18:2n6t	0.6	0.6	0.4	0.0	0.1	0.0
C18:2n6c	1.9	0.8	0.5			
C18:3n6				5.8	3.5	2.0
C18:3n3	2.6	0.5	0.3			
C20:0	0.4	0.5	0.3	0.8	0.2	0.1
C20:1	2.2	1.3	0.7			
C20:5	3.8	2.2	1.3	0.2	0.3	0.2

SD, standard deviation; SE, standard error.

## Discussion

### Food algal condition on sexual reproduction

Sexual reproduction of *D. magna* was more successful when diatom was provided. Improved behavioral character (i.e., mating frequency and copulation duration) was observed in the STE group. Sexual reproduction is a very expensive behavior because there is a trade‐off between survival and reproduction (Ferrari and Hebert 1982; Girdner and Larson 1995; Innes 1997; Shelly and Kennelly 2002). Relationships between sexual reproduction and food quality (or provision of supplemental energy sources) have been reported in *Daphnia* by Koch et al. (2011) and Abrusán et al. (2007), and other organisms including guppies (Kodric‐Brown 1989) or fruit flies (Shelly and Kennelly 2002), and they commonly emphasize the quality of food for success of sexual reproduction.

It is known that *D. magna* individuals obtain a majority of fatty acids from their food sources, and long‐chain PUFAs cannot be synthesized de novo from low‐molecular‐weight precursors (Becker and Boersma 2005). If food quality or availability does not suffice, they start to consume stored fatty acids and the consumption rate increases when egg bearing starts. Young zooplankton individuals (approximately 8 days old) contained a large amount of long‐chain PUFAs, which gradually decreased as they age and reproduce (Barata et al. 2005). This indicates that long‐chain PUFAs are necessary for somatic and population growth of *D. magna*. This is in full accordance with Ahlgren et al. (1990) who reported microalgae containing large amounts of PUFA, such as EPA and docosahexaenoic acid (DHA) allowed daphniid to grow well. From comparisons of fatty acid composition (see Table 2), we can assume that more proportion of long‐chain PUFAs resided in *S. hantzschii* would be responsible for daphniid sexual reproduction success (i.e., higher mating frequency, copulation duration, and hatching rates). This diatom is known to have >C20 PUFAs (von Elert 2002), particularly EPA and DHA (Weers and Gulati 1997). Unfortunately elemental stoichiometry (C:N:P ratio) of *S. hantzschii* is not reported, a systematically close species, *S. minutulus*, is 109:16:2.3 (Lynn et al. 2000).

Although *C. vulgaris* is a more popular microalgal food for daphniids and possesses diverse fatty acids (Ötleş and Pire 2001; Yusof et al. 2011), sexual reproduction was not much successful compared with the diatom species. The amount of long‐chain PUFAs of *C. vulgaris* was relatively smaller than *S. hantzschii*, which would be one possible cause for the low hatching rates of resting eggs. Furthermore, from stable isotope analysis, despite provision of similar amount of two algal cells, relatively higher assimilation rate was found from *D. magna* that consumed *S. hantzschii*. Assuming that rates of material absorption or digestion taken place in a *D. magna* gut are not dependent on algal species (i.e., absorption or digestion probability is equal between the algal species), *prima facie*, long‐chain PUFAs of diatom would be more absorbed by *D. magna*.

### Hatching success of male mating

Greater hatching rates of resting eggs were found from the diatom treated group in both repetitive mating experiments, which was similar to simple mating experiment. As sexual females were fed on only *C. vulgaris*, the influence of algal food treatments could be clearly observed. Similar to simple sexual reproduction, males consumed diatom might accumulate more long‐chain PUFA in their bodies, which seemed to result in amendment of various sexual reproduction characteristics.

From experimental or observational research regarding sexual mating behavior of geese, crickets, or guppies, it is found that more viable males were selected as partners at higher probability, after intra‐sexual competition (i.e., increase of male mating success rate; Kodric‐Brown 1989; Alatalo et al. 1991; Garcia‐Gonzalez and Simmons 2010). In most cases, viabilities of males are primarily affected by food availability and quality. Supplementary diet to sufficient food resulted in an increase of mating advantage for wild‐type Mediterranean fruit flies *Ceratitis capitata* (Shelly and Kennelly 2002; Shelly et al. 2002). A similar pattern was also found in the Mormon cricket, *Anabrus simplex,* in that diet control negatively affected male mating success (Gwynne 1993). Thus, the quality of food consumed by male individuals would be a crucial for determination of male mating success, which supports the current results. In contrast to those species, daphniids are believed not to possess any mechanism for attracting females; therefore, we expect that that harvested energy sources from nutritious food algae might directly increase sexual activity. The recent report has emphasized that sexual reproduction behavior of male *D. magna* flexibly responded to food availability (La et al. 2014). This implies that nutrition (or energy incorporation) of *D. magna* from microalgae is an important factor for controlling its sexual reproduction.

One of our interests focuses on the difference of hatching rates among different food algal sources. The more high‐quality food algae, the more successful hatching rate of resting egg. It can be thought that improved hatching success would be related to longer copulation duration. It may assure efficient insemination (see Ueno 1994), which would lead to higher fertilization probability. Although we found a significant relationship between body size and penis diameter, it is still unclear any biochemical mechanism related to penis shape changes resides in *D. magna*, or if so, it was activated by consumption of diatom. Increase of penis diameter would be a consequence of body size increase. However, when length of penis is not different, thicker sexual organ would be advantageous to copulation. One point to which we pay more attention is the possibility of prolonged hatching time from MIX or CHL groups. Resting eggs from STE group were successfully hatched within the period we examined; however, the eggs in the other two groups might have hatched if we had allowed more time for hatching. This point should be further explored experimentally.

### Ecological relevance

Winsor and Innes (2002) reported that *Daphnia* species are known not to conduct sexual selection for mating, but to do a random contact (sexing would occur; La et al. 2014). Some research discovered different migration patterns between males (generally horizontally moves) and females (generally vertically moves), which is thought to improve encounter rates (Watt and Young 1994). From this point of view, male *Daphnia* may require more energy to attain successful sexual reproduction, and sufficiently ingested energy from high‐quality food source leads to a successful discover of sexual females.

One point regarding induction of sexual reproduction remains in a biochemical domain, with respect to amino acid contents. It is known that low temperatures and shortage of food induce sexual reproduction in daphniids (Enserink et al. 1990; Kleiven et al. 1992), which is thought to be linked to health of the daphniids’ population. In some research (D'abramo 1979; Ahlgren et al. 1990; Ravet and Brett 2006; Abrusán et al. 2007; Koch et al. 2009), nutrition of cladocerans are important for somatic growth and reproduction. Besides, a series of algal food amino acid research have revealed that daphniids’ sexual reproduction was related with provision of several amino acids through food consumption, such as arginine and histidine (Koch et al. 2009, 2011; Fink et al. 2011). Those research emphasized that foods sources (e.g., *Scenedesmus* or *Chlamydomonas*) that did not support the required amino acids for daphniids’ metabolism tended to induce sexual females with ephippium, while high‐quality food algae provision (e.g., *Cryptomonas*) maintained subitaneous development. In this study, induction sexual females and males were based on *C. vulgaris,* thus it was not allowed to identify whether *S. hantzschii* have contributed to the initiation of sexual reproduction process. The influence of diatom food quality regarding induction of sexual reproduction relationship, and identification of specific molecules should be further addressed.

Different from parthenogenetic mode, sexual reproduction necessarily requires males. Many studies regarding sexual reproduction have emphasized the enormous cost of maintaining sex and have attempted to find the significance of sex maintenance from recovering problems of genetic mutation (Kodric‐Brown and Brown 1987; Agrawal 2001; Siller 2001). Genetic recombination, by means of sexual reproduction, also provides a chance for diversifying genetic traits to the future generation, which allows the population to overcome possible environmental stress that acts as selection pressure. We expect that this advantage can be maximized when the males experience larger proportions of high‐quality food. Practically, males that were privileged by improved food conditions (i.e., more energy source supply) showed relatively higher mating success, resulting in larger fecundity (Kodric‐Brown 1989; Kaspi et al. 2000; Shelly and Kennelly 2002). The current study also supports this idea, that is, the outcome of sexual reproduction is also affected by food quality. A further experimental approach, evidencing the influence of food quality in relation with food quantity, is necessary for this hypothesis, and supplementation of specific fatty acid or biochemical molecule (von Elert 2002; Ravet et al. 2003; Martin‐Creuzburg et al. 2010) is also a straightforward method in understanding which would be responsible for sexual reproduction characteristics. Nevertheless, it is apparent that there is a minimal role of food quality in sexual reproduction of *D. magna*.

## Conclusion

We observed the responses of sexual reproductions of *D. magna* to three different feeding conditions: provision of the diatom species *Stephanodiscus hantzschii* only (STE), the green alga *Chlorella vulgaris* only (CHL), and a mixture of the two algal food species (MIX). Compared with CHL and MIX, frequency of mating and duration of copulation significantly increased in STE group. Males’ body size and its penis diameter increased in diatom treatment, and hatching rates of resting eggs was likewise increased. Furthermore, repeated mating experiments revealed that hatching rates were greater in the diatom‐provided experimental group. Analyses of fatty acid composition of algal food sources and their contribution to the grazer by stable isotope signature detection supported the findings. The results of this study revealed that well‐nourished male individuals by provision of diatom food had high potential to promote successful fertilization of resting eggs via morphological amendment and active mating behavior.

## Conflict of Interest

None declared.

## Supporting information


**Data S1.** Sexual female and male *D. magna* induction protocol.
**Table S1.** Size and density of two food algal species used in the experiment.
