# Supplementary material for: Effects of algal food quality on sexual reproduction of Daphnia magna
Source: Ecol Evol. 2016 Mar 21;6(9):2817–32. doi: 10.1002/ece3.2058 (PMC4863008; doi:10.1002/ece3.2058)
Supplement: Supplementary file 1 — Data S1. Sexual female and male D. magna induction protocol. Table S1. Size and density of two food algal species used in the experiment. [file ECE3-6-2817-s001.docx]

**Supplementary data 1.** Sexual female and male *D. magna* induction protocol.

*Sexual female* D. magna *preparation*

We induced males and sexual females carefully from parthenogenetic maternal individuals. Birth time-synchronized (within 12 hours) *D. magna* neonates (approximately 2,000 individuals) were collected at Day 1 and reared until Day 7 under a low population density regime (500 individuals per 7,000-mL glass tank; total four tanks). The tanks were filled with 5,000 mL Elendt M4 medium, and they were placed in a plankton growth chamber (Eyela FLI-2000, Japan) under incubation conditions of 20°C and a 12L:12D light-dark cycle. We supplied sufficient *C. vulgaris* (2.5 mg C L^-1^) while daily medium exchange was implemented during this period. At Day 8, adult female individuals in the tanks were put into a high-density environment (egg-bearing and time-synchronized; approximately 2,000 adult females per 5000 mL medium) for male production. Neonates from the first clutch were observed at Day 10, and were all discarded. We collected the neonates from the second clutch at Day 12 to obtain sufficient individuals for the experiment. The collected neonates (a mixture of males and females) were divided into three groups and maintained at low density (500 individuals per 5,000 mL medium). At this stage, we applied different food treatments: STE, CHL, and MIX; each with an algal density of 2.5 mg C L^-1^ until individuals could be sexed visually (ca. 2–3 days were required; i.e., Days 14 to 15). At Day 15, we identified females from mixed group to remove them, and the females were discarded. Seven to eight additional days were spent to obtain adult male *D. magna* (i.e., Days 22 to 23) under different algal food treatment. As we summarized, the females used for male induction were fed only with *C. vulgaris*, whereas the male individuals were fed with different algal foods.

*Sexual male induction*

Induction protocol of sexual females was similar to that of male production, but there were a few differences. The conditions of the culture and initial density (i.e., 500 individuals per 5,000 mL medium; total of four tanks) were identical until Day 9. The first clutch was formed at Day 10, and we allowed the maternal *D. magna* to produce neonates from the second clutch (Day 12). These neonates were incubated for an additional 10 days under low density conditions (500 individuals per 5,000 mL medium) until they became adults. At Day 15, we removed male *D. magna* from the stock every single day for seven days, and at Day 22 we increased the density (2000 individuals per 5,000 mL medium) without exchange of medium (food was supplied; *C. vulgaris* 2.5 mg C L^-1^) for three days. This stress induced sexual females with well-developed ovaries and immature, pale, white ephippium on their carapace at Day 25. We selected these sexual females for the mating experiment, and prepared three groups including only sexual females for further experiments (fed on *S. hantzschii* only for the STE group, *C. vulgaris* only for the CHL group, and the mixture for the MIX group; see the following subsection). To synchronize the induction timing of male and sexual female *D. magna*, we introduced sexual females two days before the introduction of males.

| Species | Size (µm) | Density (cells mL^-1^) |
| --- | --- | --- |
| *C. vulgaris* | 4.03±0.9 | 6295.5±1357.2 |
| *S. hantzschii* | 4.29±0.7 | 996.3±149.1 |

**Supplementary table S1. Size and density of two food algal species used in the experiment**
